# Comparative transcriptome analyses revealed different heat stress responses in high- and low-GS *Brassica alboglabra* sprouts

**DOI:** 10.1186/s12864-019-5652-y

**Published:** 2019-04-04

**Authors:** Rongfang Guo, Xingru Wang, Xiaoyun Han, Wenjing Li, Tao Liu, Bingxing Chen, Xiaodong Chen, Gefu Wang-Pruski

**Affiliations:** 1 Joint FAFU-Dalhousie Lab, College of Horticulture, Fujian Agriculture and Forestry University, Fuzhou, 350002 China; 2 College of Horticulture, Fujian Agriculture and Forestry University, Fuzhou, 350002 China; 3 Department of Plant, Food, and Environmental Sciences, Faculty of Agriculture, Dalhousie University, Truro, NS B2N 5E3 Canada

**Keywords:** *Brassica alboglabra*, Glucosinolate biosynthesis, Heat stress, Transcriptome sequencing, HSP90

## Abstract

**Background:**

Chinese kale (*Brassica alboglabra*) contains high nutritional elements and functional molecules, especially anticarcinogenic and antioxidant glucosinolates (GS), which was highly affected by environment temperature. To investigate the link of GS biosynthesis with heat stress response in Chinese kale, global transcription profiles of high-GS line (HG), low-GS line (LG), high-GS line under heat stress (HGT) and low-GS line under heat stress (LGT) were analyzed.

**Results:**

Based on three biological replicates of each RNA sequencing data, 3901, 4062 and 2396 differentially expressed genes in HG vs HGT, LG vs LGT and HGT vs LGT were obtained, respectively. GO annotation, KEGG pathway analysis and a comprehensive analysis of DEGs showed a strong correlation between the GS biosynthesis and heat stress response. It was noticed that 11 differentially expressed genes tied to the GS biosynthesis were down-regulated, 23 heat shock transcription factors and 61 heat shock proteins were up-regulated upon the heat treatment. Another two Chinese kale varieties Cuibao and Shunbao with high- and low- GS content respectively, were used to validate the relationship of GS content and heat-response, and the results showed that high-GS content variety were more thermotolerant than the low-GS content one although GS significantly decreased in both varieties under heat stress. In addition, *HSP100/ClpB*, *HSP90*, *HSP70* and *sHSPs* were differentially expressed in high- and low-GS varieties. Notably, *HSP90* and *sHSPs* showed an obviously early response to heat stress than other related genes.

**Conclusion:**

The higher heat resistance of high-GS Chinese kale and the sharp decrease of glucosinolate content under heat stress indicated a strong relationship of GS accumulation and heat stress response. Combined with the previous report on the low expression of *HSP90* at elevated temperatures in GS-deficient mutant *TU8* of *Arabidopsis*, the differential expression pattern of *HSP90* in high- and low- GS varieties and its early heat response implied it might be a key regulator in GS metabolism and heat-resistance in Chinese kale.

**Electronic supplementary material:**

The online version of this article (10.1186/s12864-019-5652-y) contains supplementary material, which is available to authorized users.

## Background

*Brassica* species, included many important crops in the *Brassicaceae* family, play an important role in agriculture and horticulture fields and contribute greatly to economies and population health worldwide. *Brassica alboglabra*, also known as Chinese kale or Kai Lan, is widely distributed in Southern China and Southeast Asia [1]. *B. alboglabra* is preferentially consumed in China owing to its tender bolting stem and leaves, good flavor and high nutritional content in anticarcinogenic compounds and antioxidants, such as total phenolics, carotenoids, and glucosinolates (GS) [2–4] .

GS also known as mustard oil glucosides, are a group of nitrogen- and sulfur-rich secondary metabolites, mainly found in the order Capparales. They can be grouped into indole, aliphatic, and aromatic GS according to the characteristic of the amino acids they are derived from, such as Trp (indole GS), Ala, Ile, Leu, Met, or Val (aliphatic GS), and Phe or Tyr (aromatic GS) [5–7]. Generally, the biosynthesis of GS include three separate phases: the elongation of aliphatic GSs chain, the formation of the core structure, and the modification of the side chain of GS [8]. In recent years, a wide variety of crucial transcriptional factors and biosynthetic genes involved in the GS metabolism and regulation of *B. oleracea* [9], *B. alboglabra* [2, 3, 10], *B. rapa* [11, 12] were identified and studied.

GSs, also affected by some abiotic stresses, have been implicated to play an important role in plant abiotic stress response. A few studies have demonstrated that the GS biosynthesis was linked to heat stress [10, 13–15]. Ludwig-Müller et al. (2000) found that the deficiency in GS metabolism of *Arabidopsis thaliana* was remarkably less tolerant upon exposure to elevated temperatures than wild-type plants. Charron and Sams (2004) discovered that high temperatures could enhance higher aliphatic GS contents of Brassicaceae vegetables. Hara et al. (2013) showed that exogenous application of isothiocyanates in *Arabidopsis* enhanced thermo-tolerance and induced the expression of HSP genes. Guo et al. (2016a) found that heat and hypoxia stresses enhanced the accumulation of aliphatic GSs and sulforaphane in broccoli sprouts.

Higher temperature, one of the most vital abiotic stresses, severely restricted plant growth and development [16]. To survive high temperatures, plants have evolved sophisticated response mechanisms to in the long-term evolution. The response mechanism was orchestrated by modifying biochemical and physiological factors at the cellular and molecular levels. The thermal tolerance involved the expression of a set of heat shock protein (HSP) genes, mediated by effector genes and various heat shock transcription factors (HSFs). HSFs, widely existed in both plants and animals, played an important role in regulation of this heat-induced transcriptional reprogramming [17]. HSPs can act as molecular chaperones, protect cells against heat damage and function in protein folding, as well as the intracellular distribution and degradation of other proteins [18].

In recent years, RNA sequencing (RNA-Seq) has becoming a popular tool for profiling gene expression and for identifying genes or molecular pathways that are differentially expressed between two or more biological conditions [19]. Recent examples [2, 20, 21] of using RNA-Seq to investigate GS metabolism and heat stress found that genes related to GS metabolism were down-regulated under heat stress in *B. napus* silique wall [21], the aliphatic GS and sulforaphane were accumulated in broccoli sprouts under the stresses of heat, hypoxia and heat plus hypoxia [10], and that the exogenous allyl-isothiocyanate treatment triggered the expression of genes related to GS metabolism, sulphate uptake and assimilation, heat stress response, oxidative stress response, elicitor perception, plant defence and cell death mechanisms [20].

In this study, the RNA-Seq was adopted to examine the transcriptome of high-GS line (HG), low-GS line (LG), high-GS line under heat stress (HGT) and low-GS line (LG) under heat stress of *B. alboglabra*. From the transcriptome, differentially expressed genes (DEGs), especially participated in GS biosynthesis and heat stress response, were identified and characterized between HG and LG sprouts. Combining the profiles and contents of GS in another two Chinese kale varieties with high- and low- GS content respectively, and quantitative real-time PCR assay of HSF and HSP genes, we revealed a strong correlation between GS biosynthesis and heat stress response.

## Results

### Analysis of RNA-Seq data in HG and LG sprouts under heat stress

RNA-Seq was used to examine the transcript profiles in both the high-GS line and low-GS line with three biological replicates at 25 °C and 42 °C, respectively. After the filtration, the reads from RNA-seq were aligned to *B. oleracea* reference genome, and the mapped ratio ranges from 76.95 to 83.21% (Table 1). Most of the total reads (the uniquely mapped ratio ranges from 71.95 to 80.86%) were uniquely mapped to the *B. oleracea* genome, whereas a small proportion was mapped to multiple locations in the *B. oleracea* genome. The mean of GC percentage and Q30 percentage are 47.70 and 88.64%, respectively. Data for untreated high-GS and low-GS line were listed in Guo et al. (2016) [2].

**Table 1:** Summary of RNA-Seq data sets

Sample name	Read Sum	Base Sum	GC (%)	Q30 (%)	Total reads	Mapped reads	Mapped ratio (%)	Uniq mapped reads	Uniq mapped ratio (%)
HGT1	26,007,774	6,552,439,073	47.56	88.43	52,015,548	40,055,158	77.01	37,421,631	71.94
HGT2	28,396,211	7,153,993,772	47.98	88.40	56,792,422	43,991,326	77.46	41,162,110	72.48
HGT3	21,529,409	5,424,260,730	47.47	88.52	43,058,818	33,913,407	78.76	32,595,867	75.70
LGT1	32,174,870	8,106,396,261	47.57	88.77	64,349,740	51,534,294	80.08	48,995,649	76.14
LGT2	24,453,389	8,106,396,261	47.52	88.57	48,906,778	37,635,233	76.95	35,665,558	72.93
LGT3	25,796,906	6,498,964,347	47.46	88.63	51,593,812	41,119,360	79.70	39,407,551	76.38

**Fig. 1:**
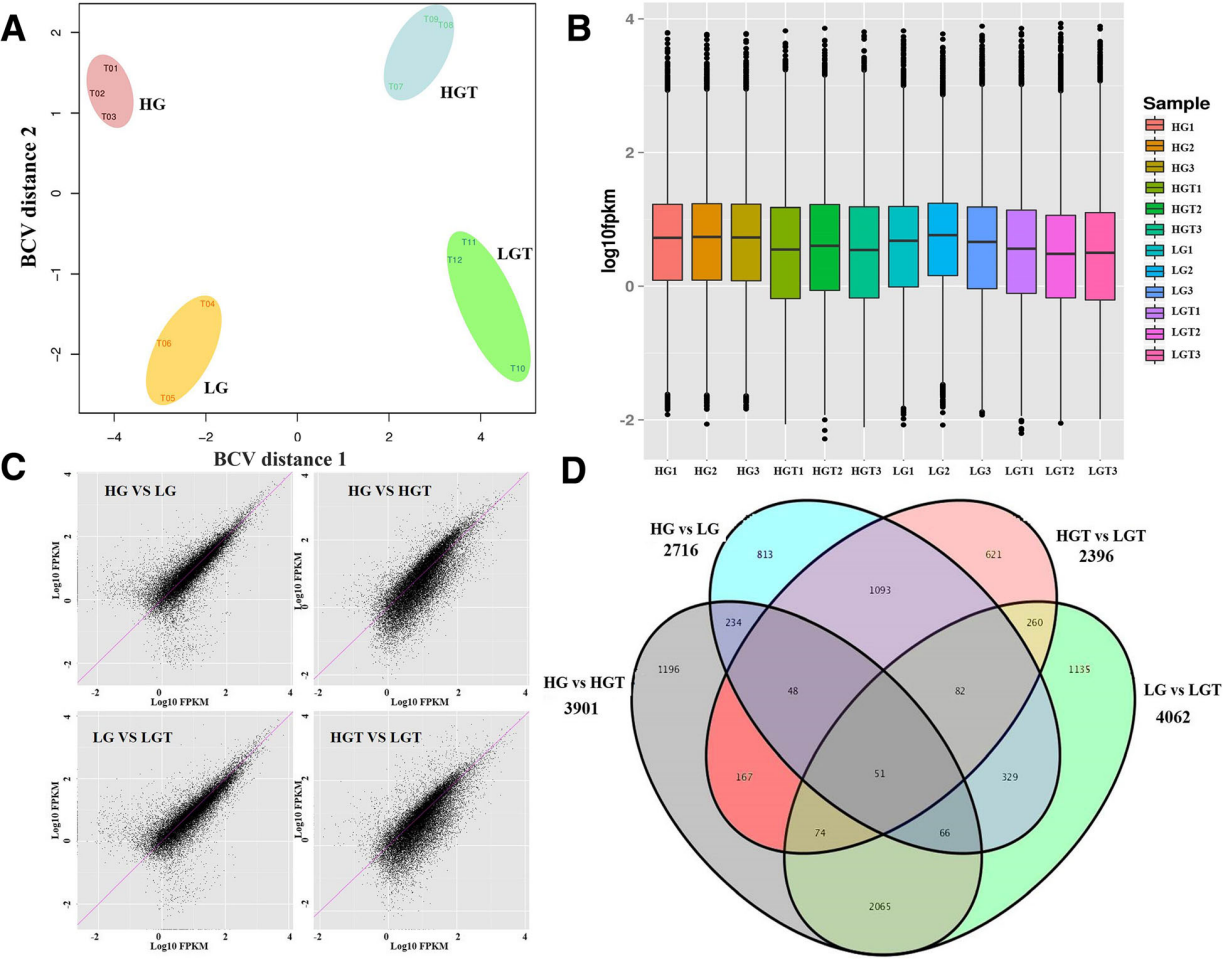
RNA-seq data qualification in HG and LG sprouts at 25 °C and 42 °C. **a** Multi-dimensional scaling (MDS) plot of RNA-seq expression profiles in two dimensions. The MDS plot shows clustering of samples based on the distance derived from biological coefficient of variation (BCV) between the paired samples. T01, T02 and T03 represent HG1, HG2 and HG3; T04, T05 and T06 represent HGT1, HGT2 and HGT3; T07, T08 and T09 represent LG1, LG2 and LG3; T10, T11 and T12 represent LGT1, LGT2 and LGT3, respectively. **b** The boxplot of overall expression levels of lines HG and LG at 25 °C and 42 °C with three biological replications, respectively. The y-axis displays the log10 (FPKM) of every sample. **c** Pairwise comparison of whole gene expression levels of lines HG and LG at 25 °C and 42 °C with three biological replications. **d** Venn diagram showing the number of DEGs in HG vs HGT, HG vs LG, LG vs LGT and HGT vs LGT, respectively

The sample homogeneity of the four groups with three replications were analyzed. RNA collected from the three replications in every sample had a low biological coefficient of variation (BCV) value, indicating that gene expression in three replications of every sample was homogeneous (Fig. 1a). This suggests that the replicated samples produced data that are acceptable for further analyses. The expression levels of 12 cDNA libraries were delineated by boxplot profiles (Fig. 1b), and a pairwise comparison of gene expression levels of the four groups was shown as Fig. 1c, showing that the samples were highly reproducible and the experimental results were reliable and credible. Gene differential expression analysis identified 8234 DEGs (log2|Fold change| ≥1 and FDR ≤ 0.01) with 2716 DEGs in HG vs LG, 3901 DEGs in HG vs HGT, 4062 DEGs in LG vs LGT and 2396 DEGs in HGT vs LGT respectively (Fig. 1d).

### Alternative splicing (AS) analysis after heat stress

**Fig. 2:**
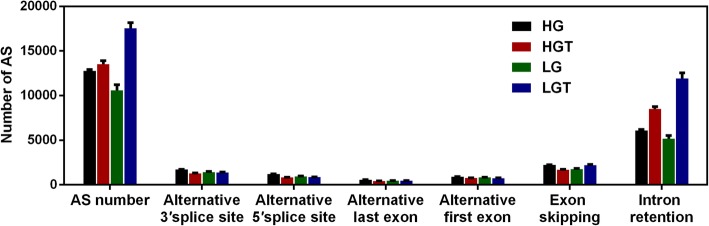
Statistical data of alternative splicing (AS) in the HG, HGT, LG and LGT. Bars represent SE from three replicates in every sample

AS events had occurred and there was an average of 13,000 AS events in every sample. AS events were classified into 6 types using soft Cufflinks, and the largest and smallest AS event groups were intron retention event and alternative last exon event, respectively (Fig. 2). The total AS events occurred in the samples germinated under heat stress were more than that in the samples germinated at normal temperature, and the statistically significant difference was mainly reflected in intron retention, suggesting that introns could play an important role in environmental responses. These results showed that the probability of AS events occurred more when circumstances changed, and that the organism could orchestrate sophisticated and refined regulatory mechanisms to respond to the altered environment and develop effective mitigation strategies.

### GO analysis of DEGs in response to heat stress

**Fig. 3:**
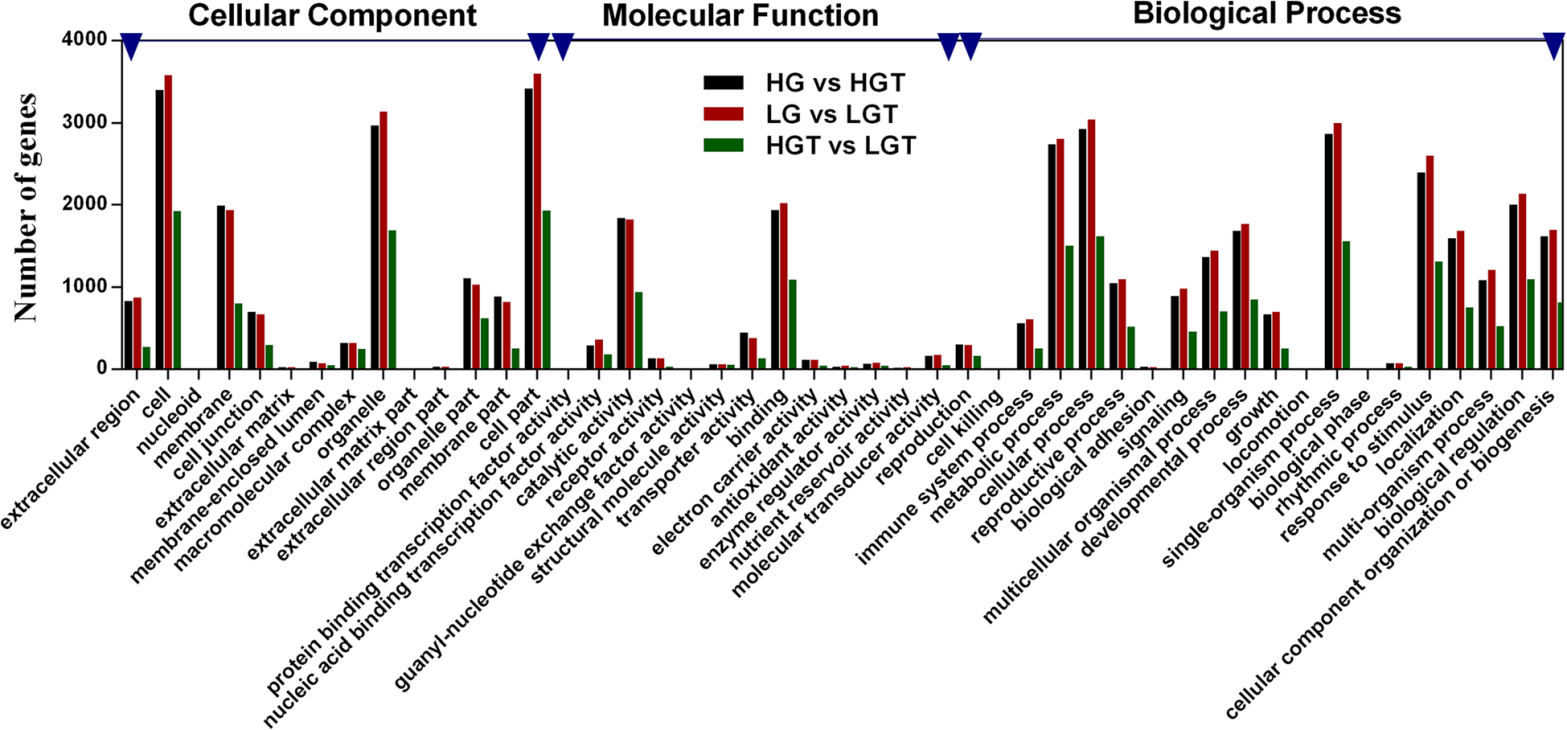
GO annotation of DEGs. GO annotation of DEGs in HG vs HGT (black column)**,** LG vs LGT (red column) and HGT vs LGT (green column). GO annotation is divided into biological process (BP), cellular component (CC) and molecular function (MF)

GO was used to analyze the functional classifications of the DEGs in HG and LG grown at 25 °C and 42 °C, respectively (Fig. 3). The DEGs were divided based on cellular component (CC), molecular function (MF) and biological process (BP). The largest CC, MF and BP subcategory for the DEGs in HG vs HGT were plasma membrane, ATP binding and response to salt stress, which comprised 33.44, 14.82 and 11.90% of the DEGs in the subcategory, respectively. This data suggested that the high-GS line underwent more exquisite metabolic activities in the plasma membrane upon response to heat stress. The largest CC, MF and BP subcategory for the DEGs in LG vs LGT were nucleus, protein binding and response to chitin, which comprised 34.69, 23.13 and 14.23% of the DEGs in the subcategory, respectively. It suggested that the low-GS line conducted more sophisticated metabolic activities in nucleus response to heat stress. Furthermore, the largest CC, MF and BP subcategory for the DEGs in HGT vs LGT were nucleus, protein binding and response to salt stress, which comprised 40.96, 21.37 and 12.27% of the DEGs in the subcategory, respectively, suggesting the different response to heat stress in HG and LG were enriched in protein functions of nucleus. In summary, these results indicated the different response mechanism between HG and LG when confronted with heat stress.

### KEGG analysis of DEGs in response to heat stress

**Fig. 4:**
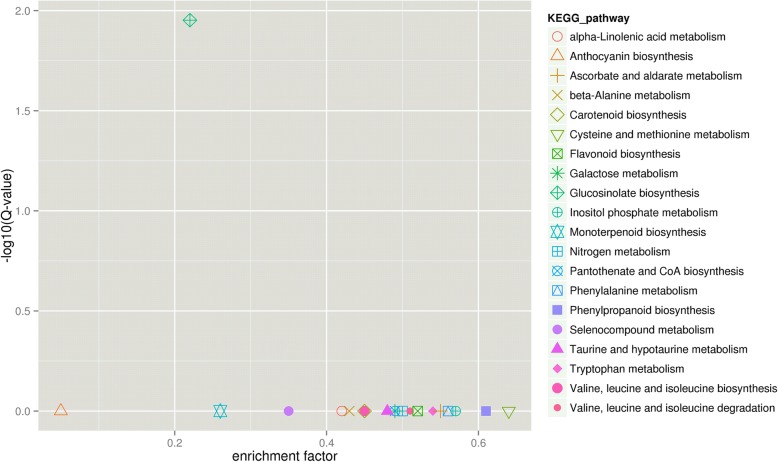
KEGG annotation of DEGs. KEGG annotation of DEGs in HGT vs LGT. Yellow column shows cellular processes, blue column shows organismal systems, purple column shows environmental information processing, green column shows metabolism, and pink column shows genetic information processing

The KEGG Pathway Database was initiated to identify the biological pathways of DEGs, and the DEGs in HG vs HGT, LG vs LGT and HGT vs LGT were found to be mainly enriched in the 104, 102 and 106 KEGG pathways, respectively. The most enriched KEGG pathway in HG vs HGT, LG vs LGT and HGT vs LGT were protein processing in endoplasmic reticulum, plant hormone signal transduction and plant hormone signal transduction, respectively (Fig. 4 and Additional file 1: Figure S1), which coincided with the results of GO analysis. Those DEGs involved in protein processing in endoplasmic reticulum related to translocon, ribosome anchor, protein recognition by luminal chaperones, N-glycan biosynthesis, re-glucosylation, de-glucosylation, lectin associated, accumulation of misfolded proteins, protein targeting, ER-associated degradation and ubiquitin ligase complex. However, compared to the DEGs in HG vs HGT, there was more DEGs in LG vs LGT and HGT vs LGT for plant hormone signal transduction related to tryptophan metabolism, zeatin biosynthesis, diterpenoid biosynthesis, carotenoid biosynthesis, cysteine and methionine metabolism, brassinosteroid biosynthesis, a-linolenic acid metabolism and phenylalanine metabolism. These changes indicated significant changes in protein modification in response to heat stress in HG, and plant hormone signal transduction in response to heat stress in LG.

**Fig. 5:**
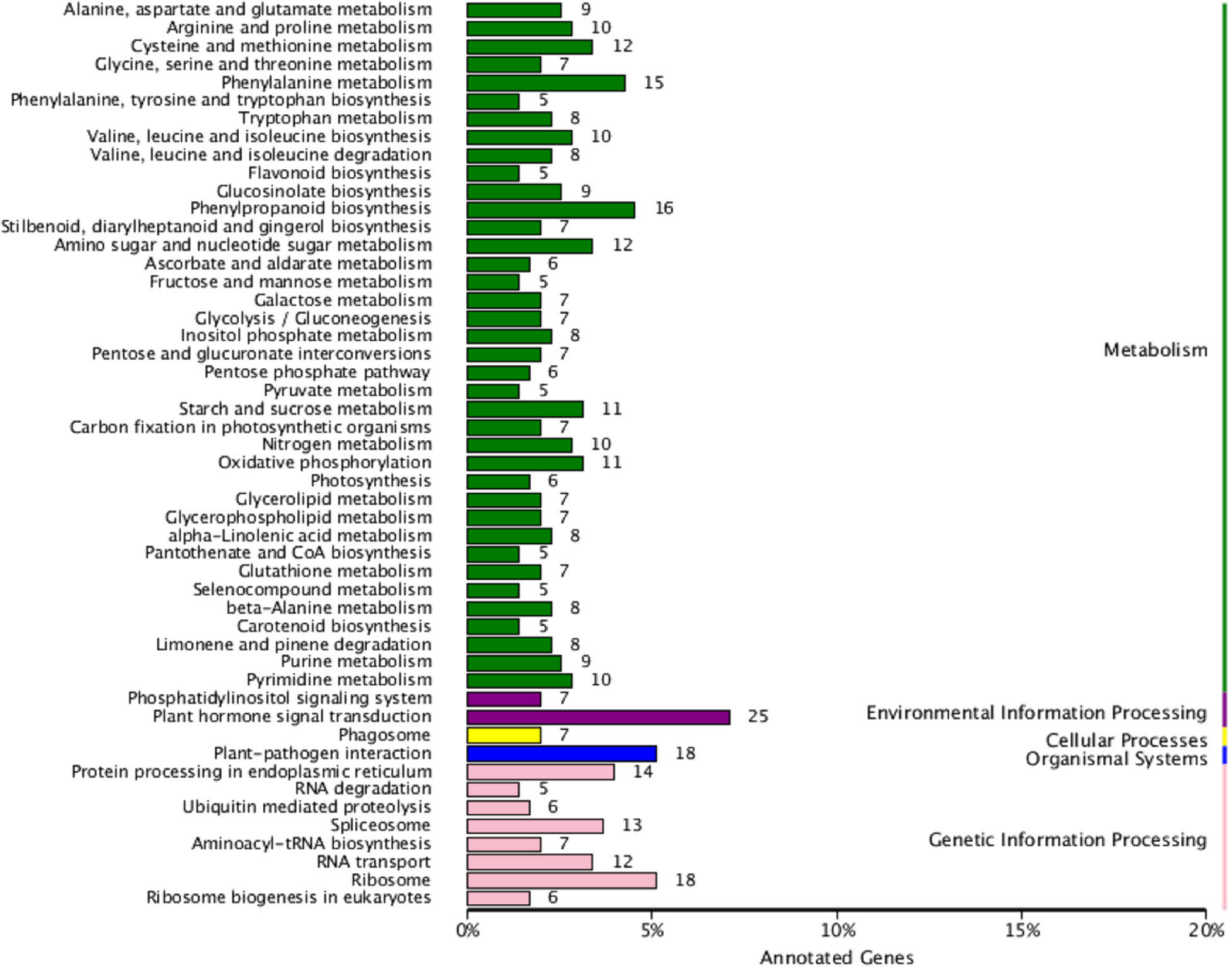
KEGG enrichment of DEGs. KEGG enrichment of DEGs in HGT vs LGT. The y-axis corresponds to different Q-value of different KEGG pathway, and the x-axis shows the enrichment factor of different KEGG pathway. The different symbols represented for different KEGG pathways

Furthermore, the significantly enriched 20 KEGG pathways in HG vs HGT, LG vs LGT and HGT vs LGT were general metabolic pathways and biosynthesis of secondary metabolites (Fig. 5 and Additional file 1: Figure S2). Of these, brassinosteroid biosynthesis, glycosaminoglycan degradation, oxidative phosphylation and phagosome uniquely represented in the HG vs HGT (Additional file 1: Figure S2A), endocylosis, ether lipid metabolism, plant hormone signal transduction and plant-pathogen interaction uniquely represented in the LG vs LGT (Additional file 1: Figure S2B). However, the significant enriched 20 KEGG pathways in HGT vs LGT were clearly different from those in HG vs HGT and LG vs LGT (Fig. 5). It should be noted that the enrichment factor of GS biosynthesis was the second smallest and its Q-value was the highest of all the KEGG pathways in HGT vs LGT, indicating that high- and low-GS Chinese kale sprouts did have different heat stress responses ascribed to the GS biosynthesis.

### Analysis of DEGs involved in GS biosynthesis

First of all, we found that 25 genes and 10 transcription factors related to GS biosynthesis altogether were differentially expressed (Tables 2 and 3), and almost all DEGs in HG vs HGT and LG vs LGT were down-regulated and accounted for the decline of GS content under heat stress. Notably, the DEGs in HG vs LG showed similar expression pattern with those in HGT vs LGT, suggesting that GS biosynthesis pathway is contributed to the differential responses between HG and LG to heat stress. This finding correlate with the results shown in Fig. 5. Together, these results demonstrated that DEGs of GS biosynthesis were down-regulated during heat stress, and that the difference of GS biosynthesis in both high- and low-GS sprouts was conducive to their different responses to heat stress during growth stage.

**Table 2:** DEGs involved in GS biosynthesis. FC is the short name of fold change of gene expression. Log2 FC1 denotes the values in HG vs HGT, Log2 FC2 denotes the values in LG vs LGT, and Log2 FC3 denotes the values in HGT vs LGT. “Inf” represented no transcript level of DEG in control sample, and “-Inf” represented no transcript level of DEG in treated sample

Category	Gene Name	Gene ID	Swissprot Annotation	Log2 FC1	Log2 FC2	Log2 FC3
Chain elongation	BCAT4	Bo3g073430	Methionine aminotransferase BCAT4	–	–	4.17
		Bo5g113720	Methionine aminotransferase BCAT4	–	–	5.22
	MAM1	Bo2g161100	Methylthioalkylmalate synthase 1, chloroplastic (Precursor)	–	–	4.61
		Bo7g098000	Methylthioalkylmalate synthase 2, chloroplastic (Precursor)	–	–	5.95
Core structure formation	CYP79F1	Bo5g021810	Dihomomethionine N-hydroxylase	–	−2.96	4.98
	CYP79B1	Bo1g002970	Cytochrome P450 79B1	–	− 2.81	–
	CYP83A1	Bo4g130780	Cytochrome P450 83A1	–	–	4.35
		Bo4g191120	Cytochrome P450 83A1	–	–	–
	SUR1	Bo7g113100	S-alkyl-thiohydroximate lyase SUR1	–	–	2.85
	UGT74B1	Bo5g041080	UDP-glycosyltransferase 74B1	2.78	–	–
	UGT74C1	Bo4g049480	UDP-glycosyltransferase 74C1	–	–	4.83
		Bo4g177530	UDP-glycosyltransferase 74C1	–	–	–
	SOT	Bo6g067810	Cytosolic sulfotransferase 1	–	–	Inf
		Bo6g097270	Cytosolic sulfotransferase 1	–	–	-Inf
		Bo6g118380	Cytosolic sulfotransferase 16	–	–	−2.51
		Bo2g080910	Cytosolic sulfotransferase 16	–	–	− 2.51
	FMO	Bo8g108370	Flavin-containing monooxygenase FMO GS-OX-like 2	–	–	− 2.11
		Bo9g028520	Flavin-containing monooxygenase FMO GS-OX-like 3	−3.98	−2.33	–
		Bo7g098490	Flavin-containing monooxygenase FMO GS-OX-like 8	–	–	2.18
		Bo9g174970	Flavin-containing monooxygenase FMO GS-OX-like 9	−2.03	–	–
	CYP81F1	Bo2g032590	Cytochrome P450 81F1	−2.32	−4.39	–
		Bo1g004730	Cytochrome P450 81F1	–	−2.48	–
		Bo9g131960	Cytochrome P450 81F1	–	−2.64	−2.72
Side chain Modification degradation	AOP2	Bo9g006220	glucosinolate GS-Alk	–	–	3.18
	TGG	Bo8g039420	myrosinase, thioglucoside glucohydrolase	–	–	3.96

Dash line “--” means there is no significant differences between treatments;

“Inf” represented no transcript level of DEG in latter sample, and “-Inf” represented no transcript level of DEG in former sample

**Table 3:** Regulatory genes involved in aliphatic GS biosynthesis that showed significant differential expression. FC is the short name of fold change of gene expression. Log2 FC1 denotes the values in HG vs HGT, Log2 FC2 denotes the values in LG vs LGT, and Log2 FC3 denotes the values in HGT vs LGT

Gene Name	Gene ID	Swissprot Annotation	Log2 FC1	Log2 FC2	Log2 FC3
MYB28	Bo5g025570	Transcription factor MYB28	–	− 3.22	−2.03
	Bo2g080900	Transcription factor MYB28	–	−2.99	–
	Bo8g104300	Transcription factor MYB28	–	−2.72	− 2.00
	Bo9g014610	Transcription factor MYB28	–	–	5.92
	Bo7g098590	Transcription factor MYB28	–	–	3.22
	Bo8g104210	Transcription factor MYB28	–	–	2.11
	Bo8g091100	Transcription factor MYB28	–	–	–
MYB29	Bo8g067910	Transcription factor MYB29	–	−3.73	−2.12
	Bo9g175680	Transcription factor MYB29	–	–	2.68
MYB76	Bo9g164230	Transcription factor MYB76	2.09	–	–

Dash line “--” means there is no significant differences between treatments;

“Inf” represented no transcript level of DEG in latter sample, and “-Inf” represented no transcript level of DEG in former sample

### Analysis of DEGs involved in heat stress response

To identify genes responsive to heat stress in high-GS and low-GS line, we analyzed the *HSFs* and *HSPs*. In total, 23 *HSFs* and 61 *HSPs* were differentially expressed in HG vs LG, HG vs HGT, LG vs LGT, and HGT vs LGT (Table 4). Overall, it also showed that FC values of most of *HSF* and *HSP* transcripts in HG were slightly lower than that in LG exposed to heat stress, which implied that low-GS *B. alboglabra* was less thermotolerant to heat stress.

**Table 4:** Expression of DEGs related to heat stress transcription factors (HSFs) and heat shock proteins (HSPs) in high- and low- GS sprouts. FC is the short name of fold change of gene expression. Log2 FC1 denotes the values in HG vs HGT, Log2 FC2 denotes the values in LG vs LGT, and Log2 FC3 denotes the values in HGT vs LGT

Category	Gene ID	Swissprot Annotation	Log2 FC1	Log2 FC2	Log2 FC3
Heat stress transcription factors, HSFs	Bo1g155010	heat stress transcription factor A1e	–	4.41	2.55
	Bo3g042080	heat stress transcription factor A2	6.85	8.00	–
	Bo1g020010	heat stress transcription factor A4a	3.09	3.29	–
	Bo7g106320	heat stress transcription factor A4a	2.41	–	–
	Bo3g157380	heat stress transcription factor A4a	2.08	3.37	–
	Bo4g190180	heat stress transcription factor A4a	–	–	–
	Bo2g165560	heat stress transcription factor A4a	–	–	8.89
	Bo4g190160	heat stress transcription factor A4a	–	–	−5.69
	Bo3g081420	heat stress transcription factor A6b	3.17	2.64	–
	Bo7g101330	heat stress transcription factor A7a	8.25	8.86	–
	Bo4g125760	heat stress transcription factor A7a	6.56	8.34	–
	Bo05169s010	heat stress transcription factor A7a	Inf	Inf	–
	Bo7g101320	heat stress transcription factor A7a	6.95	Inf	–
	Bo8g098580	heat stress transcription factor A7b	4.64	4.70	–
	Bo2g056350	heat stress transcription factor A8	–	2.06	–
	Bo7g118320	heat stress transcription factor B1	3.81	4.22	–
	Bo1g005040	heat stress transcription factor B1	3.59	4.18	–
	Bo3g108110	heat stress transcription factor B2a	3.29	5.87	–
	Bo2g161820	heat stress transcription factor B2a	7.08	7.70	–
	Bo3g045370	heat stress transcription factor B2b	2.87	5.00	–
	Bo3g035750	heat stress transcription factor B3	2.90	–	− 2.26
	Bo6g008100	heat stress transcription factor B4	–	–	–
	Bo3g083520	heat stress transcription factor C1	–	− 2.05	–
Heat shock proteins, HSPs	Bo8g044750	14.7 kDa heat shock protein	3.92	–	–
	Bo3g163960	15.4 kDa class V heat shock protein	5.38	2.37	–
	Bo5g007530	17.2 kDa class II heat shock protein	2.75	2.22	–
	Bo3g184440	17.4 kDa class III heat shock protein	5.34	6.19	–
	Bo3g007430	17.6 kDa class II heat shock protein	5.97	7.56	–
	Bo2g010790	17.6 kDa class II heat shock protein	4.47	5.41	–
	Bo4g061450	17.6 kDa class I heat shock protein 2	3.74	3.98	–
	Bo4g169420	17.6 kDa class I heat shock protein 2	2.47	4.66	8.22
	Bo3g130430	17.6 kDa class I heat shock protein 3	3.30	7.19	8.42
	Bo2g025510	18.1 kDa class I heat shock protein	7.35	8.04	–
	Bo3g017800	18.1 kDa class I heat shock protein	4.64	6.17	–
	Bo7g006050	18.5 kDa class IV heat shock protein	4.69	3.77	–
	Bo6g048040	22.0 kDa heat shock protein (Precursor)	4.26	4.36	–
	Bo1g039850	23.6 kDa heat shock protein, mitochondrial (Precursor)	5.61	6.49	–
	Bo1g050780	25.3 kDa heat shock protein, chloroplastic (Precursor)	5.28	6.48	–
	Bo6g028160	26.5 kDa heat shock protein, mitochondrial (Precursor)	5.43	5.65	–
	Bo8g007140	peroxisomal small heat shock protein ACD31.2	–	–	3.80
	Bo1g037840	heat shock 70 kDa protein 6, chloroplastic (Precursor)	2.57	3.76	–
	Kale newGene	heat shock 70 kDa protein 7, chloroplastic (Precursor)	−2.55	–	–
	Bo1g053470	heat shock 70 kDa protein 17 (Precursor)	−2.71	–	–
	Bo1g078880	heat shock 70 kDa protein 17 (Precursor)	–	–	-Inf
	Bo03352s010	heat shock protein 90	–	3.93	2.93
	Bo9g108350	heat shock protein 90–1	–	2.85	–
	Bo2g037660	heat shock protein 90–2	2.68	3.88	–
	Bo3g021230	heat shock protein 90–2	3.04	3.90	–
	Bo5g048850	heat shock protein 90–2	–	–	3.79
	Bo3g020890	heat shock protein 90–2	2.05	2.84	–
	Bo32505s010	heat shock protein 90–3	–	3.87	–
	Bo13090s010	heat shock protein 90–4	–	5.34	4.59
	Bo1g126250	chaperone protein ClpB1	–	–	−2.57
	Bo2g105000	heat shock protein STI	4.29	5.76	–
	Bo8g102330	DnaJ protein homolog (Precursor)	4.84	5.71	–
	Bo9g183160	DnaJ protein homolog (Precursor)	−2.29	–	–
	Bo1g017440	DnaJ protein homolog (Precursor)	–	2.29	–
	Bo8g054350	DnaJ protein homolog (Precursor)	–	3.93	3.65
	Bo1g023930	DnaJ protein homolog (Precursor)	−2.95	–	–
	Bo7g113150	DnaJ protein homolog 2 (Precursor)	2.97	4.03	–
	Bo6g079480	DnaJ protein homolog 2 (Precursor)	−2.66	–	2.94
	Bo4g098740	DnaJ protein homolog 2 (Precursor)	−3.17	−2.49	–
	Bo4g098750	chaperone protein dnaJ 2 (Precursor)	3.04	4.94	–
	Bo9g149730	chaperone protein dnaJ 3 (Precursor)	2.12	2.76	–
	Bo6g112630	chaperone protein dnaJ 6	5.55	7.97	–
	Bo1g134560	chaperone protein dnaJ 6	2.14	3.52	–
	Bo00581s030	chaperone protein dnaJ 6	3.40	2.94	–
	Bo5g133600	chaperone protein dnaJ 6	−2.26	–	–
	Bo5g131620	chaperone protein dnaJ 6	3.02	6.86	–
	Bo2g095520	chaperone protein dnaJ 8, chloroplastic (Precursor)	–	−2.51	–
	Bo8g101690	chaperone protein dnaJ 10	–	2.00	–
	Bo9g026330	chaperone protein dnaJ 11, chloroplastic (Precursor)	–	−4.30	–
	Bo7g097830	chaperone protein dnaJ 11, chloroplastic (Precursor)	4.54	4.43	–
	Bo1g005990	chaperone protein dnaJ 11, chloroplastic (Precursor)	–	−2.14	–
	Bo1g138440	chaperone protein dnaJ 11, chloroplastic (Precursor)	–	2.21	–
	Bo6g115880	chaperone protein dnaJ 13	5.25	5.27	–
	Bo4g088200	chaperone protein dnaJ 15	–	–	–
	Bo9g084660	chaperone protein dnaJ 20, chloroplastic (Precursor)	−2.47	–	–
	Bo5g022100	chaperone protein dnaJ 39	−2.31	–	–
	Bo5g139610	DNAJ heat shock N-terminal domain-containing protein	2.83	4.97	–
	Bo9g004530	DNAJ heat shock N-terminal domain-containing protein	–	−2.81	–
	Bo3g052770	DNAJ heat shock N-terminal domain-containing protein	–	−2.84	–
	Bo4g004510	DNAJ heat shock N-terminal domain-containing protein	–	−3.46	− 2.20
	Bo1g144350	posttranslational modification, chaperones	–	–	−2.42
	Bo5g118640	heat shock protein -related	−2.05	−2.19	2.54

Dash line “--” means there is no significant differences between treatments;

“Inf” represented no transcript level of DEG in latter sample, and “-Inf” represented no transcript level of DEG in former sample

In plants, there were 3 classes in the HSF protein families, classes A, B and C, which were discriminated by peculiarities of their flexible linkers and HR-A/B regions [22]. In this study, 6 class A, 4 class B and 1 class C were differentially expressed under heat shock stress, and the detailed fold change (FC) values of these HSF members were provided (Table 4). Four interesting features were worth mentioning. First, the 3 members (Bo4g190180, Bo2g165560 and Bo4g190160) of class A4a group and one class B4 were found to be constitutively expressed and were not up-regulated by heat stress, therefore, it is speculated that they were generally present in an inactive form. Secondly, in contrast to the expressions of classes B and C, the expressions of class A were different and diverse in response to heat stress, suggested that the class A was paraphyletic and HSFA genes were functionally characterized extensively. Thirdly, the *A7*, *B1* and *B2a Hsf* genes with highly expressed under heat stress were likely to be important HSF members for keeping the expression of *Hsp* genes at a high level. Lastly, *HsfC1* was down-regulated in response to heat stress, which was different from *HSFA* and *B*, indicating distinct regulational modes of *HSFs* during heat stress response.

HSPs were divided into five conserved classes: HSP100/Clp, HSP90, HSP70, HSP60 and the small heat-shock proteins (sHSPs) [23]. In this work, one *ClpB1*, 8 *HSP90s*, 4 *HSP70s* and 17 *sHSPs* were identified (Table 4). All *sHSPs* (except *Bo8g007140*) were up-regulated expressed in response to heat stress to endow cells with thermotolerance. Notably, the expressions of two *HSP17.6* (*Bo4g169420* and *Bo3g130430*) in line JL09 were higher than in JL08 under same condition, suggesting that some *HSP17.6* in JL09 may be constitutively expressed at relatively high levels and more susceptible to high temperature induction. However, the expressions of *HSP90* and *HSP70* were intricate compared with sHSPs: three *HSP70s* in the same line were up- or down-regulated expressed induced by heat stress, the expression of *HSP70* (Bo1g078880) and *ClpB1* were not influence by heat shock, which were similar with *HSF4a* (*Bo4g190180*, *Bo2g165560,* and *Bo4g190160*), while all *HSP90* were up-regulated but the expression of *HSP90* in HG and LG were differently induced by heat stress. Furthermore, we found that the co-chaperone of HSP70, *DnaJ*, was also differentially expressed under heat shock conditions.

### Different heat response in HG and LG Chinese kale sprouts under heat stress

**Fig. 6:**
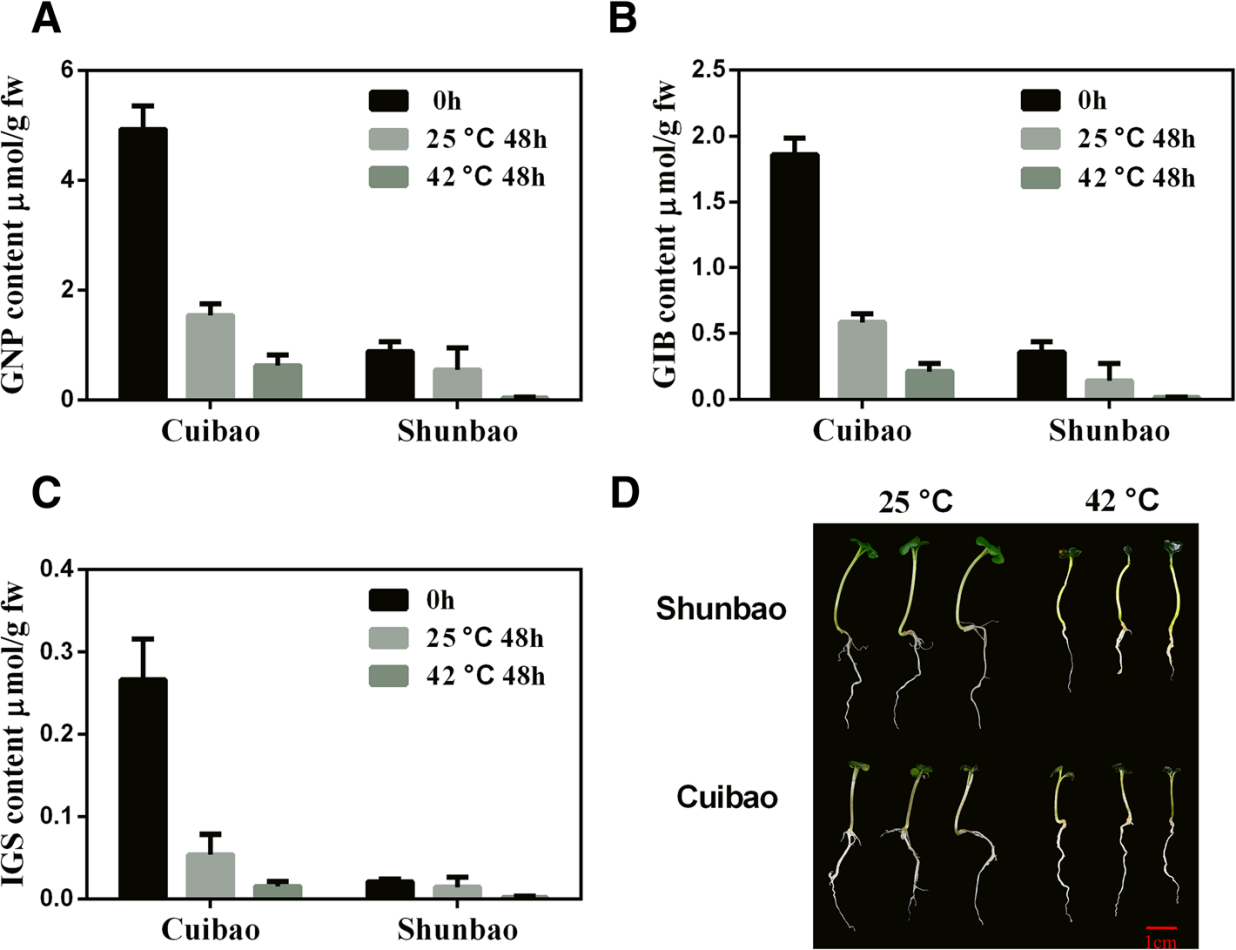
GS content and growth characteristic of Cuibao and Shunbao under heat stress. **a** GNP (Gluconapin), **b** GIB (Glucoiberin) and **c** IGS (Indole GS) content of Cuibao and Shunbao after treated with 42 °C for 48 h, respectively. The bars represented the standard deviation from three independent experiments with three replications in each experiment. **d** Growth characteristic of Cuibao and Shunbao after treated with 42 °C for 72 h

Another two Chinese kale varieties Cuibao and Shunbao were used to validate the differential heat-stress response in high- and low- GS content sprouts. To examine the GS diversity of both lines, we applied HPLC method to compare the GS profile and the content. As expected, Cuibao and Shunbao sprouts mainly contained aliphatic GS including gluconapin (GNP) and glucoiberin (GIB), and a small quantity of indole GS (IGS). As well, the initial GS contents (0 h) of Cuibao were significantly higher than those of Shunbao (Figs. 6a, b and c). The GS contents (GNP, GIB and IGS) of Cuibao and Shunbao were sharply reduced when grown at 42 °C for 48 h compared to the control (Figs. 6a, b and c), suggesting that GS contents in both lines o were decreased by heat stress. It was also found that after treated with heat stress for 72 h Cuibao showed more thermotolerance than Shunbao (Fig. 6d). These results indicated that high-GS content variety was more resistant to heat stress than low-GS variety.

### Expression pattern of heat-resistant related genes in high- and low- GS sprouts

**Fig. 7:**
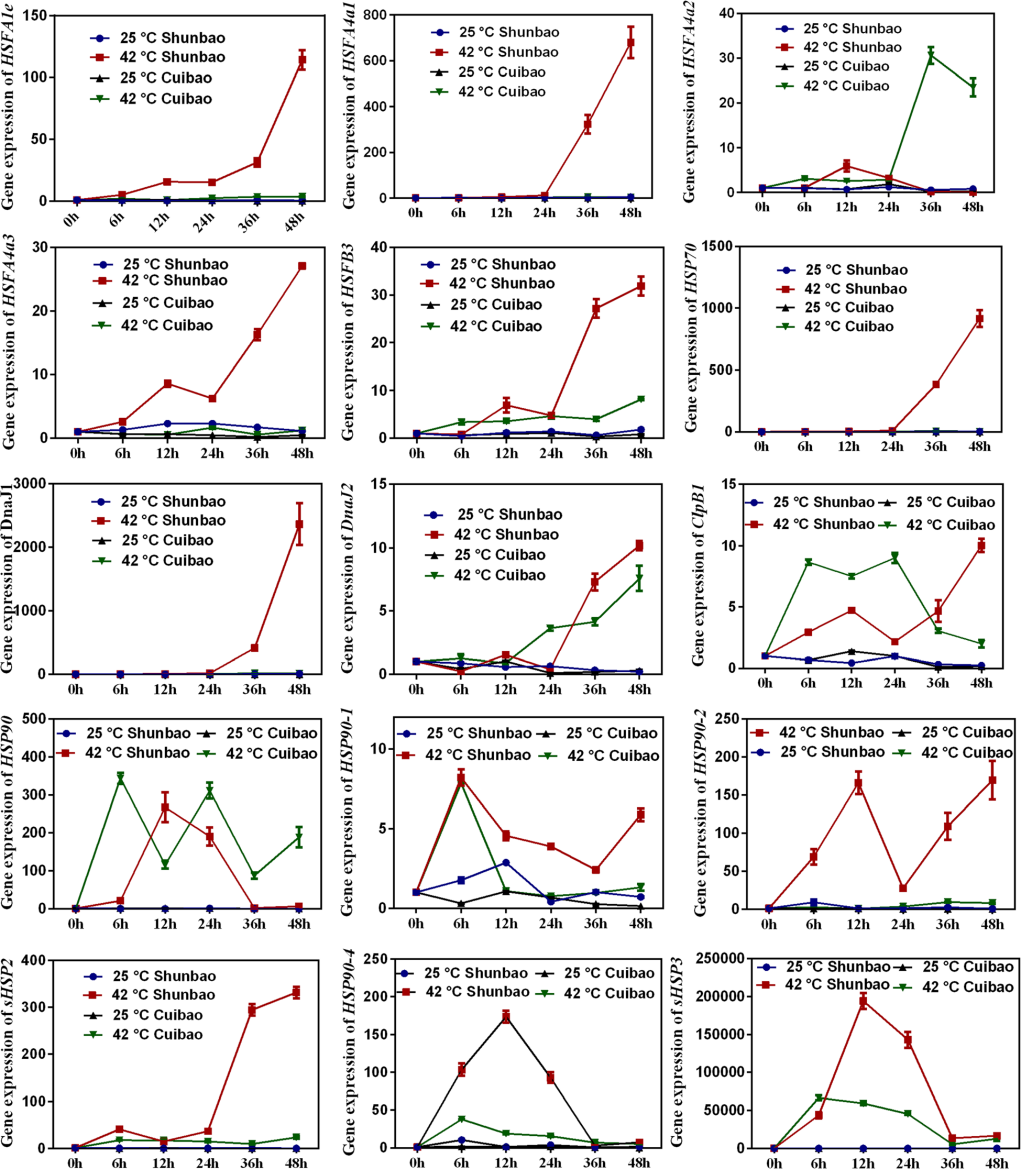
Expression of *HSFs* and *HSPs* genes in Cuibao and Shunbao under heat stress. The *actin2* gene was used as an internal control to normalize the expression data. The bars represent the standard deviation from three independent experiments with three replicates each

To investigate the expression pattern of *HSFs* and *HSPs* in high- and low- GS sprouts, a time course of expression of these genes was performed (Fig. 7). Most of the genes showed relatively high expression in Shunbao at 42 °C relative to Cuibao at 42 °C with little to no major changes over time, except for *HSF4a2*, *DnaJ2*, *ClpB1*, and *HSP90*. Especially, the expression of *HSFA4a1* was delayed compared with *HSFA1e*, *HSFA4a2*, *HSFA4a3,* and *HSFB3* in Shunbao at 42 °C. The expressions of *HSP70*, *DnaJ1*, *DnaJ2* and *sHSP2* in Shunbao and Cuibao were later than HSFs, suggesting the regulation of *HSF* on some of *HSPs*. However, the expressions of *ClpB1* and all *HSP90s* in Shunbao and Cuibao were rapidly activated in heat treatment, except for *HSP90–2* in Cuibao, indicating that there were some HSF-dependent pathways for activated expression of *HSPs* in response to heat stress.

## Discussion

With global warming growing more severe, heat stress is becoming an agricultural problem in many areas in the world. Heat stress has a significant adverse impact on plant growth and development, which leads to agricultural productivity worldwide [24]. Thus, the molecular mechanism of the heat stress response and breeding of heat-tolerant plants is necessary to protect food production and ensure crop safety. To survive high temperatures, many heat-responsive transcription factors and genes are induced to protect plants from heat shock stress [25]. Since the GS contents of *B. alboglabra* sprouts greatly changed, this study was initiated with the aim to explore possible crosstalk between GS metabolism and heat shock stress response.

In this work, we performed comparative studies to assess the effects of exposure heat stress of high- and low-GS content sprouts at the transcriptomic levels. Our particular interest was the identification of DEGs involved in GS metabolism in response to heat stress, which could help explain the regulatory mechanism of GS metabolism under heat stress.

### GS accumulation under heat stress condition

The mechanism of GS accumulation under high temperatures has not been conclusive, because most of the earlier studies focused on higher temperatures as a way to improve GS contents in *Brassica* sprouts or cruciferous products. In sprouts, the change of GS contents under high temperatures is increased upon heat treatments. The 7-day old to 11-day old broccoli sprouts grown under three day/night temperature regimes (30 °C /15 °C, 22 °C /15 °C, and 18 °C /12 °C) showed decreased contents of glucoraphanin and total GS contents under higher temperature condition (30 °C /15 °C) [26]. When higher temperature (60 °C) were used to treat broccoli sprouts every 24 h, the aliphatic GS including GRA, GNA, and GER in 3-day, 5-day, and 7-day old sprouts were significantly increased [10]. In plants, it is proven that the concentration of GS in *B. oleracea* leaves and stems were higher at 32 °C while at 22 °C the GS in roots decreased [13]. The application of GS degradation products ITCs including phenethyl ITC, methyl ITC, and allyl ITC can mitigate the growth inhibition induced by heat stress at 55 °C for 1 h in *Arabidopsis*. The pre-administration of phenethyl ITC increased the expression level of heat tolerance related *heat shock protein (HSP) 70* [15].

The thermal degradation of GS was first demonstrated in synthetic glucobrassicin [27]. After that, cooking method including boiling, microwaving and canning were used to study the effect of temperature on GS reduction. Severe heat treatments like canning were found to result in 73% degradation of GS in red cabbage [28–30]. The kinetic modeling of different kinds of GS degradation in broccoli and Chinese kale after treated by a heating block at 100 °C were established in which it was found that the GS degradation rates were related to their structure and the kinetic parameters that was affected by planting season [31]. The heat treatment for broccoli sprouts powder was carried out at 130 °C and the results showed that GS with different structures has different degradation rates. The methylsulfinyl GS (glucoiberin, glucoraphanin and glucoalyssin) are more stable than the methylsulfanyl GS (glucoiberverin and glucoerucin) and among the methylsulfinyl GS a longer chain (glucoalyssin) results in increased thermal breakdown [32]. It is ambiguous how high temperature affects the GS in *Brassica* plants. Mutant experiment showed that the expression of *HSP 90* were reduced at elevated temperatures in GS-deficient mutant *TU8* of *Arabidopsis* [14], indicating a potential relation between high temperatures and GS biosynthesis. In our study, the GS content was decreased dramatically and the expression of genes related to GS biosynthesis were down-regulated in Cuibao and Shunbao after treated with the high temperature (42 °C) for 48 h. This is consistent with the reduction of GS related gene expressions in heat-treated *Brassica napus*’ silique wall at the seed-filling stage [21]. However, in low-GS variety JL-09, the expression of GS biosynthetic genes was up-regulated and sulfur metabolism genes were down-regulated compared with the high-GS variety JL-08 under 42 °C condition, indicating that under heat stress condition, the metabolic flux in low-GS content variety were orienting GS biosynthesis. Maybe the amount of GS is related to the heat response.

### Comparison of the transcriptional response in different GS-content Chinese kale sprouts under heat stress

HSFs are the terminal components of signal transduction and mediate the expression of HSPs responsive to both heat stress and a large number of chemical stressors [22]. In plants, there are 3 classes in the HSF protein family (classes A, B, and C). Among these, HSF A genes generally serve as bona fide transcription factors binding to heat shock element (HSE) that are found in *Arabidopsis* [33]. HSF B members have been demonstrated to act as repressors or co-activator of class A HSFs and were also capable of binding to HSE [34–36]. Yet knowledge about the biological role of the HSF B and HSF C members was scarce. However, individual HSF members had species-specific features in regulating genes involved in the heat stress response [37]. In *Lycopersicon esculentum*, there are at least 25 HSF protein families. HSF A1a acted as a master regulator for triggering the heat response and acquired thermo-tolerance [38], and HSF B1 functions as a transcription co-activator, cooperating with class A HSFs during heat stress [39]. Whereas, there are 21 HSF family members in *Arabidopsis thaliana* and no master regulator has been found [40]. Furthermore, three subclass A1 HSFs (A1a, A1b, and A1d) in *A. thaliana* were functionally redundant for triggering the heat response [41, 42], and HSF B1 was found to act as a repressor [34–36, 43].

In this work, 23 *HSFs* were expressed differentially in *B. alboglabra* sprouts. It was shown that the expressions of *HSF* genes in Chinese kale were significantly up-regulated by heat shock stress, suggesting commonalities of HSFs in the heat stress response in these two lines. Unexpectedly, we found that all HSFs except HSFB4 interacted with HSP70 (Bo1g037840), HSP90 (Bo03352s010, Bo2g037660, Bo3g020890, Bo3g021230, Bo5g048850, Bo32505s010, Bo13090s010) and chaperone protein dnaJ (Bo8g102330, Bo1g017440, Bo7g113150, Bo9g149730, Bo1g134560, Bo00581s030, Bo5g131620, Bo6g112630, Bo1g005990, Bo6g115880, Bo5g139610), but no interactions between HSFs. These analyses seemingly suggest that 1) no master regulator, transcription co-activator or repressor in *B. alboglabra*, which was different from *L. esculentum* and *A. thaliana*; 2) HSFs in *B. alboglabra* functioned redundantly, as in the case of the *Arabidopsis* Hsf A1 subfamily [41, 44–46]; 3) HSF C member was a transcriptional repressor of *HSP* genes, which has not been shown in any other plant species. Importantly, it showed that the expression patterns of *HSF A1e*, *HSF A4a* (*Bo7g106320*), *HSF A8*, *HSF B3*, *HSF B4* and *HSFC1* were different in high- and low- GS varieties, indicating the different heat shock response between high- and low-GS *B. alboglabra* sprouts under heat stress.

HSP families, including HSP100/ClpB, HSP90, HSP70, HSP60 and sHSPs, regulated by HSFs, can be induced to respond to an array of physiological and environmental stresses. HSP protect cells from degradation, oxidative stress, hypoxia and thermal stress by catalyzing the refolding of damaged or denatured proteins to prevent protein aggregation and promote protein disaggregation [47, 48]. The action mode of HSFs and HSPs in tomato has been widely researched. The HSF and HSP networks in plants were controlled at the transcriptional level by cooperation of distinct HSF members and by interaction with chaperones. In response to heat shock stress, HSFs not only regulated HSP genes expression, but also were concomitantly subjected to cell-type-dependent feedback regulation through factor-specific physical and functional interactions with chaperones belonging to HSP90, HSP70 and small HSP families [49]. Hahn et al. (2011) found that HSP70 and HSP90 regulated HSF function by direct interactions in tomato, HSP70 repressed the activity of HSF A1, HSP90 affected the abundance of *HSF A2* and HSF B1 by modulating *HSF A2* transcript degradation involved in regulation of the timing of HSF A2 synthesis [50].

In this study, *HSP100/ClpB*, *HSP90*, *HSP70* and *sHSPs* were differentially expressed and the expression levels of most *HSPs* in high-GS variety were different from those in low-GS variety, indicating the different actions of HSPs when the two lines exposure to heat stress. It is worth to note that *HSP90* was expressed prior to some *HSFs*, indicating there were some HSF-independent pathways for activated expression of HSP in response to heat stress.

## Conclusions

Taken together, this study confirmed the presence of the following transcription features: 1) down-regulation of pathways may either serve as a passive adaptation or an active resistance/protection measure to save energy or reduce consumption, such as, the down-regulated GS pathway under heat shock stress; and 2) the up-regulation of a subset of specific heat-response genes could prompt or rapidly induce a protective process upon heat shock.

## Methods

### Plant materials

Four kinds of Chinese kale (*B. alboglabra*) including JL08, JL09, Cuibao and Shunbao were used in this study. Seeds were germinated in sterile petri dishes with sterile filter paper and 15 mL of sterile water after disinfected in 0.7% sodium hypochlorite for 30 min, then drained and washed seven times with distilled water. The germinated seedlings were transferred to another sterile petri dish with sterile filter paper. One hundred seedlings and 10 mL sterile water was added per dish every three days after day one. All plants were grown in 16 h day/8 h night at 25 °C for 5 days and then 42 °C for 2 days, respectively. The lines JL08 were of high-GS contents, named as HG, and JL09 were of low-GS contents, named as LG. After treated with high temperature (42 °C), HG and LG were abbreviated as HGT and LGT respectively. JL08 and JL09 were cultivated in the greenhouse of College of Horticulture in Fujian Agricultural and Forestry University and used for transcriptome sequencing as they have been self-crossed for five generations as described in Guo et al. (2016) [2]. To validate the relationship of heat-resistance and GS content in Chinese kale sprouts, another two varieties with different GS content and heat resistance ability, Cuibao and Shunbao (Produced by the Musashino Seed Co., LTD. Tokyo, Japan), were used for GS profiling, content assays and heat resistant related gene assays. Fujian Agriculture and Forestry University has approved the study. The land accessed is not privately owned and protected. No protected species were sampled. No specific permissions were required for these locations/activities.

### Measurement of GS content

GS were extracted and analyzed as previously described [51, 52]. Samples (500 mg) were boiled in 3 mL water for 10 min. After transferring the supernatant to a new tube, the residues were washed with 3 mL distilled deionized water (ddH_2_O), and the combined aqueous extract was applied to a DEAE-Sephadex A-25 (30 mg) column (pyridine acetate form) (Sigma, St. Louis, MO, USA). The column was washed three times with 20 mM pyridine acetate and twice with ddH_2_O. The GS were converted into their desulpho analogues by overnight treatment with 100 μL of 0.1% (1.4 units) aryl sulphatase (Sigma, St. Louis, MO, USA) added into the column, and the desulpho GSs were eluted with 2 × 0.5 mL water. HPLC analysis was performed using an HPLC system consisting of an Agilent HPLC series chromatograph (Agilent Technologies). A Hypersil C18 column (5 μm particle size, 4.6 mm × 250 mm, Elite Analytical Instruments Co., Ltd., Dalian, China) was used with a mobile phase of acetonitrile and water at a flow rate of 1.0 mL/min. The procedure employed isocratic elution with 1.5% acetonitrile for the first 5 min; a linear gradient to 20% acetonitrile over the next 15 min followed by isocratic elution with 20% acetonitrile for the final 10 min. A 40 μL sample was injected into the column by an autosampler. Absorbance was detected at 226 nm. Ortho-nitrophenyl-β-d-galactopyranoside (Sigma, St. Louis, MO, USA) was used as an internal standard for HPLC analysis. Desulphoglucosinolates were identified by comparison of retention time and quantified by peak area. The GS concentration was expressed as μmol/g fresh weight (fw) of sprouts.

### RNA extraction, RNA-Seq library construction and sequencing

Total RNA was extracted from 7-day old Chinese kale sprouts of lines JL-08 and JL-09 using the TRIzol reagent (Invitrogen, Carlsbad, CA) according to the manufacturer’s instructions. The purity and concentration of the total RNA was detected by NanoDrop 1000 spectrophotometer (Thermo Fisher Scientific, Wilmington, DE, USA) and Qubit® 2.0 Flurometer (Life Technologies, CA, USA), respectively. The integrity of the RNA samples was assessed using the RNA Nano 6000 Assay Kit of the Agilent Bioanalyzer 2100 system (Agilent Technologies, CA, USA). The purified RNA was dissolved in RNase-free water and stored at − 80 °C.

The construction of RNA-seq library was performed following procedures similar to previous study [2, 53]. Briefly, after enrichment and purification with oligo (dT)-rich magnetic beads, the mRNA was cleaved into short fragments. Then first- and second-strand cDNA were synthesized using the mRNA fragments as templates. The cDNA was purified by AMPure XP beads and resolved with elution buffer for end reparation and single nucleotide adenine addition, and the short fragments were connected with adapters. Then the suitable fragments were selected as templates for PCR amplification. Finally, RNA-seq libraries were sequenced using an Illumina HiSeq™ 2500 at Biomarker Technologies Corporation (Beijing, China) and submitted to the SRA database (PRJNA427496).

### RNA-Seq reads mapping and transcript assembly

After sequencing, raw reads containing sequencing adapters and low-quality reads were removed. *Brassica oleracea* genome and gene model annotation files were directly downloaded from the genome website (http://plants.ensembl.org/Brassica_oleracea/Info/Index). Index of the reference genome was constructed using Bowtie [54], and paired-end clean reads were aligned to the reference genome using Top Hat 2 (http://ccb.jhu.edu/software/tophat/index.shtml) [55] with all parameters set to their default values. Then the Sequence Alignment Map (http://samtools.sourceforge.net) (Li et al., 2009) files were generated by Top Hat2 and subsequently transcripts were assembled by Cufflinks (http://cufflinks.cbcb.umd.edu) [56].

### Transcriptome annotation, novel transcript prediction, and alternative splicing analysis

To annotate the Chinese kale genes, the sequences were compared to Gene Ontology (GO) [57], Kyoto Encyclopedia of Genes and Genomes (KEGG) [58], Swiss-Prot [59] and the NCBI non-redundant protein sequence databases by using the BLASTx algorithm with an *e*-value threshold of 10^− 5^ [60]. Prior to differential gene expression analysis for each sequenced library, the read counts were adjusted using the Edge R program package through one scaling normalized factor. Sequence splicing and redundancy was determined by DESeq [61]. The DEGs were identified from the Fragments Per Kilobase of transcript per Million fragments mapped (FPKM) values using the DEGseq R package [61]. The resulting *P* values were adjusted using the Benjamini and Hochberg’s approach for controlling the false discovery rate (FDR). Genes with an adjusted *P*-value < 0.01 found by DESeq were assigned as differentially expressed, and the FDR < 0.01 and |log2 (fold change)| ≥1 was set as the threshold for significantly differential expression.

The Cufflinks v2.1.1 Reference Annotation Based Transcript assembly method was used to construct and identify both known and novel transcripts from Top Hat alignment results. Alternative splicing (AS) events were classified into 6 basic types by Asprofile v1.0. The number of AS events in each sample was estimated.

### Hierarchical clustering, GO, and KEGG analysis

The overall gene expression patterns were separately analyzed based on the expression levels of the DEGs in lines JL-08 and JL-09, respectively. GO enrichment analysis (http://www.geneontology.org) of the DEGs was implemented by the GOseq R packages [19], and the corrected P-value < 0.05 was set as the threshold for GO analysis. Enrichment analysis of DEGs in the KEGG (http://www.genome.jp/kegg/) biological pathway was conducted, and we used KOBAS software [62] to test the statistical enrichment of DEGs in KEGG pathways.

### Gene expression validation

Real-time quantitative RT-PCR (qRT-PCR) analyses were performed with independent samples from lines JL-08 and JL-09 grown at 25 °C and 42 °C with the same conditions as those used for RNA-Seq analysis. The cDNAs were reversely transcribed using PrimeScript™ RT reagent Kit with gDNA Eraser (Takara, Otsu, JP), and qRT-PCR analyses were performed on an CFX96 Touch™ Real-Time PCR Detection System (Bio-Rad Laboratories, Inc.) using SYBR® Premix Ex Taq™ II (Tli RNaseH Plus) (Takara, Otsu, JP). The *Actin* gene from *B. alboglabra* was used as an internal control to normalize the expression data. Gene expression was evaluated by the 2^-ΔΔCt^ method [63]. The gene-specific primers are listed in Additional file 1: Table S1.

## Additional file

Additional file 1: **Table S1.** Primers used in the verification of differential expressed genes in high- and low-GS Chinese kale sprouts under heat stress. **Figure S1.** (A) KEGG annotation of DEGs in HG vs HGT. (B) KEGG annotation of DEGs in LG vs LGT. Yellow column shows cellular processes, blue column shows organismal systems, purple column shows environmental information processing, green column shows metabolism, and pink column shows genetic information processing. **Figure S2.** (A) KEGG enrichment of DEGs in HG vs HGT. (B) KEGG enrichment of DEGs in LG vs LGT. The y-axis corresponds to different Q-value of different KEGG pathway, and the x-axis shows the enrichment factor of different KEGG pathway. The different symbols represented for different KEGG pathways. (DOCX 767 kb)

## Abbreviations

AS: Alternative splicing
BCV: Biological coefficient of variation
BP: Biological process
CC: Cellular component
ddH_2_O: distilled deionized water
DEGs: Differentially expressed genes
FDR: False discovery rate
FPKM: Fragments per kilobase of transcript per million fragments mapped
fw: fresh weight
GIB: Glucoiberin
GNP: Gluconapin
GO: Gene ontology
GS: Glucosinolates
HG: High-GS line
HGT: High-GS line treated with heat stress
HSF: Heat shock transcription factors
HSPs: Heat shock proteins
IGS: Indole GS
KEGG: Kyoto encyclopedia of genes and genomes
LG: Low-GS line
LGT: Low-GS line treated with heat stress
MF: Molecular function
qRT-PCR: Real-time quantitative RT-PCR
RNA-Seq: RNA sequencing
