# Supplementary material for: Comparative transcriptome analyses revealed different heat stress responses in high- and low-GS Brassica alboglabra sprouts
Source: BMC Genomics. 2019 Apr 4;20:269. doi: 10.1186/s12864-019-5652-y (PMC6450006; doi:10.1186/s12864-019-5652-y)
Supplement: Supplementary file 1 — Table S1. Primers used in the verification of differential expressed genes in high- and low-GS Chinese kale sprouts under heat stress. Figure S1. (A) KEGG annotation of DEGs in HG vs HGT. (B) KEGG annotation of DEGs in LG vs LGT. Yellow column shows cellular processes, blue column shows organismal systems, purple column shows environmental information processing, green column shows metabolism, and pink column shows genetic information processing. Figure S2. (A) KEGG enrichment of DEGs in HG vs HGT. (B) KEGG enrichment of DEGs in LG vs LGT. The y-axis corresponds to different Q-value of different KEGG pathway, and the x-axis shows the enrichment factor of different KEGG pathway. The different symbols represented for different KEGG pathways. (DOCX 767 kb) [file 12864_2019_5652_MOESM1_ESM.docx]

**Comparative Transcriptome Analyses Revealed Different Heat Stress Responses in High- and Low-GS *Brassica alboglabra* Sprouts**

Rongfang Guo^a, b^, Xingru Wang^a^, Wenjing Li^a^, Xiaoyun Han^a^, Tao Liu^a^, Jingjing Wu^a^, Bingxing Chen^b^, Xiaodong Chen^b^, Faxiu Lan^a^, Gefu Wang-Pruski^a, c *^

^a^Joint FAFU-Dalhousie Lab, College of Horticulture, Fujian Agriculture and Forestry University, Fuzhou 350002, China;

^b^College of Horticulture, Fujian Agriculture and Forestry University, Fuzhou 350002, China

^c^Department of Plant, Food, and Environmental Sciences, Faculty of Agriculture, Dalhousie University, Truro, NS B2N 5E3, Canada.

**Additional files**

Table S1. Primers used in the verification of differential expressed genes in high- and low-GS Chinese kale sprouts under heat stress.

Figure S1. (A) KEGG annotation of DEGs in HG vs HGT. (B) KEGG annotation of DEGs in LG vs LGT. Yellow column shows cellular processes, blue column shows organismal systems, purple column shows environmental information processing, green column shows metabolism, and pink column shows genetic information processing.

Figure S2. (A) KEGG enrichment of DEGs in HG vs HGT. (B) KEGG enrichment of DEGs in LG vs LGT. The y-axis corresponds to different Q-value of different KEGG pathway, and the x-axis shows the enrichment factor of different KEGG pathway. The different symbols represented for different KEGG pathways.

**Table S1. Primers used in the verification of differential expressed genes in high- and low-GS Chinese kale sprouts under heat stress.**

| Gene name | Forward primer | Reverse primer |
| --- | --- | --- |
| BaClpB1 | TTCATCCAAGGGAAGCGA | CCTATGCTCTCCGTATCTGACA |
| BaDnaJ1 | TGCCACTTCCTACTGCTGCT | TTTCACCAAACGAGGCGT |
| BaDnaJ2 | TTGGTGGTGGTGGTTCAA | TGGGCATTTGTCACAGACC |
| BaHSFA1e | TCGTTCTTAGCGAAGGCTG | CCGTGATTGTTGGTATGCG |
| BaHSFA4a1 | TGATTCAGTTGTCTCGTGGG | TGATTCTGGCAAGTTCTGGA |
| BaHSFA4a2 | CAAGTTTGGGCTTAGCACG | AGATAGACTCCTGGCGGCTT |
| BaHSFA4a3 | TGAGTTCCTGGGAGATGTTT | CTGGAGAAATCATTATGCCC |
| BaHSFB3 | CACACTCCCACTCACATAACAA | CGTCGTCGTCTTCTTCTCCT |
| BaHSP70 | CTCCAACTGATAATCGTATGCC | GGTTACCATCTTCTCCGTCC |
| BaHSP90 | TCCCAGAGTGAGAACGACA | AGGTCTAAGTCTTCCTTGCTGA |
| BaHSP90-2 | CGAGTGGGATTTGGTGAAC | TGATAAAGACACGGCGGA |
| BaHSP90-4 | TGACCAGCCTCAAGGATTAC | CCTCGGACTCTTCCAGTTTC |
| BasHSP2 | GGACTTGCCTGGACTCAAA | GACATCAGCCTTCTTGGTCTC |
| BasHSP3 | AAGTGGACTGGAGGGAGACA | TGACCGACAACACACCGTT |
| BaACD31.2 | TCGGCAAGCATCTTCTGA | GCAAAGGCTGTTCCGTAGA |

Figure S1





Figure S2
